# Molecular study of drought response in the Mediterranean conifer *Pinus pinaster* Ait.: Differential transcriptomic profiling reveals constitutive water deficit‐independent drought tolerance mechanisms

**DOI:** 10.1002/ece3.6613

**Published:** 2020-08-31

**Authors:** Nuria de María, María Ángeles Guevara, Pedro Perdiguero, María Dolores Vélez, José Antonio Cabezas, Miriam López‐Hinojosa, Zhen Li, Luís Manuel Díaz, Alberto Pizarro, José Antonio Mancha, Lieven Sterck, David Sánchez‐Gómez, Célia Miguel, Carmen Collada, María Carmen Díaz‐Sala, María Teresa Cervera

**Affiliations:** ^1^ Departamento de Ecología y Genética Forestal Centro de Investigación Forestal (CIFOR) Instituto Nacional de Investigación y Tecnología Agraria y Alimentaria (INIA) Madrid Spain; ^2^ Unidad Mixta de Genómica y Ecofisiología Forestal Instituto Nacional de Investigación y Tecnología Agraria y Alimentaria (INIA)/Universidad Politécnica de Madrid (UPM) Madrid Spain; ^3^ Centro de Investigación en Sanidad Animal (CISA‐INIA) Madrid Spain; ^4^ Departamento de Cultivos Herbáceos Centro de Investigación Agroforestal de Albaladejito Cuenca Spain; ^5^ Ghent University Department of Plant Biotechnology and Bioinformatics Ghent Belgium; ^6^ VIB‐UGent Center for Plant Systems Biology Ghent Belgium; ^7^ Bioinformatics Institute Ghent Ghent University Ghent Belgium; ^8^ Departamento de Ciencias de la Vida Universidad de Alcalá Alcalá de Henares Spain; ^9^ BioISI‐Biosystems & Integrative Sciences Institute Faculdade de Ciências Universidade de Lisboa Lisboa Portugal; ^10^ Instituto de Biologia Experimental e Tecnológica (iBET) Oeiras Portugal; ^11^ Grupo de investigación Sistemas Naturales e Historia Forestal UPM Madrid Spain

**Keywords:** differential transcript profiles, Mediterranean conifer, pre‐adapted genotypes, response strategies, water stress

## Abstract

Adaptation of long‐living forest trees to respond to environmental changes is essential to secure their performance under adverse conditions. Water deficit is one of the most significant stress factors determining tree growth and survival. Maritime pine (*Pinus pinaster* Ait.), the main source of softwood in southwestern Europe, is subjected to recurrent drought periods which, according to climate change predictions for the years to come, will progressively increase in the Mediterranean region. The mechanisms regulating pine adaptive responses to environment are still largely unknown. The aim of this work was to go a step further in understanding the molecular mechanisms underlying maritime pine response to water stress and drought tolerance at the whole plant level. A global transcriptomic profiling of roots, stems, and needles was conducted to analyze the performance of siblings showing contrasted responses to water deficit from an ad hoc designed full‐sib family. Although *P. pinaster* is considered a recalcitrant species for vegetative propagation in adult phase, the analysis was conducted using vegetatively propagated trees exposed to two treatments: well‐watered and moderate water stress. The comparative analyses led us to identify organ‐specific genes, constitutively expressed as well as differentially expressed when comparing control versus water stress conditions, in drought‐sensitive and drought‐tolerant genotypes. Different response strategies can point out, with tolerant individuals being pre‐adapted for coping with drought by constitutively expressing stress‐related genes that are detected only in latter stages on sensitive individuals subjected to drought.

## Graphical abstract

The manuscript presents the transcriptome of maritime pine trees that show contrasted response to drought. Comparison of transcriptomic profiles at the organ level allowed to identify organ‐specific and genotype‐specific transcripts as well as differentially expressed genes between organs, genotypes, and treatments. The results also point out to different response strategies, with tolerant plants pre‐adapted for coping with drought by expressing constitutively stress‐related genes that only can be detected in sensitive plants submitted to drought.

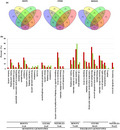

## 1 INTRODUCTION

Long‐living forest trees have developed a unique capacity to respond to environmental changes. Their adaptation and survival under adverse conditions is dependent on essential traits, such as those ones related to drought tolerance. As water deficit increases, stomata begin to close (even at moderate water deficits) as a mechanism to reduce water losses, which limits CO_2_ uptake and hence reduces photosynthetic activity (Chaves & Oliveira, 2004). Moreover, physiological mechanisms associated with drought and heat stresses may limit drastically productivity and directly cause tree mortality (Allen et al., 2010). According to recent climate projections, water deficiency will be a recurrent and increasingly acute problem in the Mediterranean area (Cook, Anchukaitis, Touchan, Meko, & Cook, 2016; Cook, Smerdon, Seager, & Coats, 2014; Dubrovský et al., 2014), which will be intensified by decrease in soil water content and evapotranspiration. In fact, paleoclimate field reconstructions estimate that 1998–2012 was the driest 15‐year period in this area since 12th century (Cook et al., 2016).

Plant responses to water stress are a complex process that is controlled by multiplex regulatory events involving signaling, ion transport, and transcription regulation to control cell turgor loss, increase in solute concentration, reduction of relative water content, and cell membranes shrinkage, which alters interactions with the cytoskeleton and cell wall (Haswell & Verslues, 2015). In response to soil dehydration, concentration of endogenous abscisic acid (ABA) progressively increases activating a cascade of physiological responses, including stomatal closure, one of the earliest plant responses (Osakabe, Shinozaki, & Tran, 2014; Polle, Chen, Eckert, & Harfouche, 2019). This compound is produced in stressed roots and transported to leaves to regulate stomatal aperture (Schachtman & Goodger, 2008). Plant stomatal closure results in lower rates of CO_2_ uptake, decreased photosynthesis rate and increased photorespiration, which lead to the accumulation of reactive oxygen species (ROS), toxic bioproducts that also act as signaling molecules (Mittler, 2017). All these changes induce synthesis of osmoprotectants to cope with osmotic stress and re‐establish osmotic homeostasis (Valliyodan & Nguyen, 2006). Groups of genes involved in biosynthesis and signaling pathways of ABA and other hormones such as auxin, ethylene, salicylic acid, jasmonic, gibberellin, and cytokinin are also involved in drought response (reviewed byUrano et al., 2017; Verma, Ravindran, & Kumar, 2017).

Current sequencing technologies improve our understanding on the genetics of complex traits allowing gene function to be monitored at the entire genome level. Transcriptomic analyses are contributing to obtain a genome‐wide view of drought response associated with different physiological changes induced in different plant species, including herbaceous and woody species (Barghini, Cossu, Cavallini, & Giordani, 2015; Bhogireddy et al., 2020; Du et al., 2018; Dugas et al., 2011; Fox et al., 2018; Iovieno et al., 2016; Kakumanu et al., 2012; Oono et al., 2014; Sakuraba, Kim, Han, Lee, & Paek, 2015). Thus, comparative transcriptomic profiling provides information about differentially expressed genes (DEGs) in contrasting conditions, different organs, and/or genotypes. Differential expression analyses have allowed identification of conserved and species‐specific genes involved in cellular homeostasis and protection from stress regulated by complex regulatory networks integrated by transcription factors (TFs), kinases, and phytohormones (reviewed by Joshi et al., 2016; Osakabe etal., 2014; Tiwari, Lata, Chauhan, Prasad, & Prasad, 2017).

Maritime pine (*Pinus pinaster*) is one of the most important forest species in the Mediterranean basin, being the main source of softwood in southwestern Europe. It is considered as a drought‐avoiding species because of its sensitive stomata and rapid osmotic adjustment in response to water deficit (Picon, Guehl, & Ferhi, 1996). However, evidences of intraspecific variation have been found in traits related to drought tolerance (Aranda, Alía, Ortega, Dantas, & Majada, 2010; Corcuera, Gil‐Pelegrin, & Notivol, 2010; de Miguel, Sanchez‐Gomez, Cervera, & Aranda, 2012; Nguyen‐Queyrens & Bouchet‐Lannat, 2003; Sánchez‐Gómez, Majada, Alía, Feito, & Aranda, 2010), as well as in drought adaptation (see also Eveno et al., 2008; Grivet et al., 2011). Molecular studies developed during the last decades have provided some additional insights. Thus, the use of cDNA‐AFLPs (Dubos et al., 2003; Dubos & Plomion, 2003) and microarrays (Perdiguero, Barbero, Cervera, Collada, & Soto, 2013; Perdiguero et al., 2012) has led to the identification of a few hundred candidate genes related to water use efficiency, including genes encoding dehydrins and genes related to cuticular wax biosynthesis. These studies found that, in general, although the variation in response to water deficit was conserved in aerial organs and roots, some genes were expressed in a time‐dependent manner in specific organs (Plomion et al., 2016). In addition, QTL mapping strategies allowed the identification of genomic regions associated with water usage efficiency containing positional candidate genes (Brendel, Pot, Plomion, Rozenberg, & Guehl, 2002; Marguerit et al., 2014; de Miguel et al., 2014). Although recurrent or severe drought periods can limit maritime pine growth (Martínez‐Vilalta & Piñol, 2002; Sabaté, Gracia, & Sánchez, 2002), molecular studies did not detect a trade‐off between water usage efficiency and growth in this pine species (Plomion et al., 2016).

The current study is focused on the genetic analysis of maritime pine drought response at the whole plant level. With this aim, we developed a transcriptomic analysis of roots, stems, and needles of siblings selected from a full‐sib cross between progenitors according to their contrasting response to drought (drought‐tolerant versus drought‐sensitive genotypes). *Pinus pinaster* is considered a recalcitrant species for vegetative propagation in adult phase (Greenwood, 1987), and therefore, most of the studies have been carried out analyzing single trees. The analysis was, however, designed using ramets (three clonal replicates of each genotype) from F_1_ progeny individuals to study general mechanisms associated with sensitive or tolerant responses in the family. We identified organ‐specific genes, genes differentially expressed in sensitive and tolerant genotypes in response to drought as well as drought‐related genes, expressed in control conditions, that may be involved in the mechanisms determining pine tolerance to hydric stress.

## 2 MATERIALS AND METHODS

### 2.1 Plant material and experimental design

Two drought‐sensitive (4, 147) and two drought‐tolerant (132, 144) genotypes were selected from Gal1056 x Oria6, a reference full‐sib family designed to study drought tolerance in maritime pine (de Miguel et al., 2012). These genotypes were vegetatively propagated as previously described in de Miguel et al. (2012). For water stress treatment, 24 2‐year‐old ramets (six clonal replicates of each genotype) were grown in a growth chamber under controlled conditions (watering to full capacity, 70% relative humidity, 20–25°C, 16/8 photoperiod, 13h of maximum light intensity at 800 μmol photons m^−2^ s^−1^) during an establishment phase of two months. Then, half of the ramets were subjected to moderate water stress. Well‐watered plants (WW plants) were kept at a VWCs (soil volumetric water content) higher than 20 vol.%, while in plants submitted to water deficit (WD plants) soil water content was depleted down to 5 vol.% in 19 days and kept at this stage for 43 additional days (Sánchez‐Gómez, Mancha, Cervera, & Aranda, 2017). At the end of drought period, roots, stems, and needles were individually harvested from each ramet and stored at −80°C.

### 2.2 RNA extraction, quality determination, cDNA library preparation, and sequencing

**FIGURE 1:**
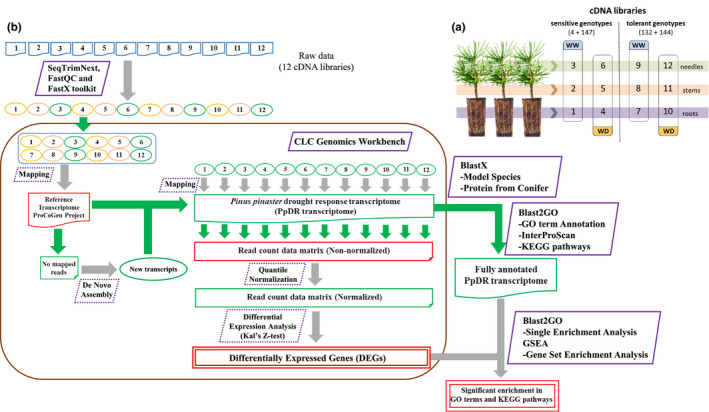
Experimental design and data analysis. (a) Experimental design. Twelve cDNA libraries were constructed and sequenced using as template RNAs extracted from different organs (roots, stems, needles) from ramets of two sensitive genotypes (4 and 147) and two tolerant genotypes (132 and 144). These genotypes were vegetatively propagated and grown under well water (WW, pools 1, 2, 3, 7, 8, and 9) and water deficit conditions (WD, pools 4, 5, 6, 10, 11, and 12). (b) Pipeline used for data analysis. Schematic overview of the pipeline used to process raw sequence files to identify constitutive and differentially accumulated transcripts. The software and files used or generated in each step are indicated

For RNA extraction, each frozen tissue sample was individually powdered in a CryoMill (Retsch) using liquid nitrogen. A total of 12 pools were made bulking equal amount of each grinded root, stem, or needle tissue from WW ramets (pools 1–3) and WD ramets (pools 4–6) of the sensitive genotypes and from WW ramets (pools 7–9) and WD ramets (pools 10–12) of the tolerant genotypes (Figure 1a). Total RNAs were extracted from needles and stems using PureLink Plant RNA Reagent Kit (Invitrogen). Extraction of total RNA from roots was performed according to Chang, Puryear, and Cairney (1993). A total of 100 μg RNA was digested with RQ1 RNase‐free DNaseI (Promega) during 30 min at 37°C and purified using the Amicon Ultra 0.5 ml (Millipore Corporation). RNA concentration and purity were estimated determining the spectrophotometric absorbance of the samples and the A260/A280 nm and A260/A230 nm ratios using at 230, 260, and 280 nm a spectrophotometer (Thermo Scientific; NanoDrop 1000) and integrity analyzed by agarose gel electrophoresis before and after DNaseI digestion. Finally, approximately 10 μg of each total RNA was further cleaned and concentrated using RNA MinElute CleanUp Kit (Qiagen) and RNA concentration was determined using the 2100 Bioanalyzer and the RNA 6000 Nano Kit (Agilent). RNA samples with an RNA integrity number (RIN) higher than 7 were used for library preparation. Additionally, using Plant/Fungi Total RNA Purification Kit (Norgen Biotek Corp.), a total of 72 RNAs were extracted from all samples, representing each of the three organs of the three WW ramets and three WD ramets of each genotype (four genotypes × two treatments × three organs × three biological replicates), to be used as template for quantitative real‐time PCR (qRT‐PCR).

Twelve cDNA libraries were constructed (Figure 1a). Sequencing was performed on a Roche Genome Sequencer FLX instrument (454 Life Sciences‐Roche Diagnostics, http://www.454.com/) at Lifesequencing S.L. Sequences were uploaded to the SRA database with BioProject ID PRJNA590115 (SRA accession numbers from SRR11812379 to SRR11812390). Each cDNA library was constructed from 1 μg cDNA using cDNA Rapid Library Preparation Kit (Roche). Preparation of cDNA libraries and emulsion PCR conditions were performed according to the Roche GS FLX manual.

### 2.3 Sequence processing and improvement of reference transcriptome

The quality of reads was checked during sequence processing with FastQC software (Andrews, 2010). Reads were trimmed and filtered removing reads with a quality score lower than 20, with similarity with common microorganisms or plastids, or low complexity reads, using SeqTrimNext software (Falgueras et al., 2010). When processed reads in a library showed overrepresented sequences or high proportion of Kmer content according to FastQC, a second trimming step rejecting 50 pb at the end of the sequences was performed using the FastX toolkit (http://hannonlab.cshl.edu/fastx_toolkit). CLC Genomics Workbench v.10 software (CLC Bio) was used for the successive steps (Figure 1b). In order to obtain an improved *P. pinaster* transcriptome, including specific transcripts from sequenced libraries which could be of interest in this analysis, reads were mapped against all the transcripts contained in the reference *P. pinaster* transcriptome (http://www.procogen.eu) available in plaza website (Proost et al., 2009). Unmapped reads were recovered and de novo assembled to identify putative transcripts higher than 200 bp, which were added to transcriptome for the successive steps.

### 2.4 Functional annotation of the generated reference transcriptome

The new reference transcriptome was re‐annotated to increase the overall total coding length and update function assignment to the new coding regions identified. All transcripts were used as query for a BlastX in two steps. Initially, all sequences were compared with RefSeq proteins database using a subset of plant model species with rich functional annotations (*Physcomitrella patens*, *Arabidopsis thaliana*, *Ricinus communis*, *Medicago truncatula*, *Glycine max*, *Oryza sativa*, *Zea mays*, *Phoenix dactyliphera*, *Malus domestica*, *Populus trichocarpa*, and *Eucalyptus globulus*) using an *E*‐value threshold of 10^–5^. Sequences without blast results followed a second round of comparison using BlastX, against a database containing all proteins encoded in gene models from fully sequenced conifer genomes (*Pinus taeda* and *P. lambartiana*; http://pinegenome.org/pinerefseq/, *Picea abies* and *Picea glauca*; http://congenie.org/) using an *E*‐value threshold of 10^–5^.

BlastX results were imported to Blast2GO, as implemented in the OmicsBox v1.2.4 software package (Conesa et al., 2005) in order to infer gene ontology (GO) terms from the different GO ontologies (biological process, molecular function, and cellular component) associated with homologous genes in model species. Additional annotations inferred by similarity to protein families, domains, and functional sites from secondary databases were obtained using InterProScan implemented in OmicsBox software. Enzyme codes and KEGG (Kyoto Encyclopedia of Genes and Genomes, Kanehisa, 2000) pathway annotation were generated in Blast2GO by direct mapping GO terms to their enzyme code equivalents. Finally, the GO annotations were filtered according to Plant GO‐Slim. Sequences, descriptions, and annotations of PpDR_transcriptome are available in the Figshare repository under the https://doi.org/10.6084/m9.figshare.12328979.

### 2.5 Mapping and differential gene expression analysis

Reads from each library were mapped to previously generated transcriptome using default scoring values using CLC Genomics Workbench v.10 (CLC Bio), ignoring the reads with multiple mapping positions. Count data matrix was normalized using quartile normalization in order to avoid differences in deep sequencing between libraries.

A first exploration of sequencing data was performed grouping samples according to organ (*n* = 4) in order to validate gene expression. A differential expression analysis using Limma package (Smyth, Ritchie, & Thorne, 2011) implemented in Babelomics suite 5.0 (Alonso et al., 2015) was performed grouping data from roots (pools 1, 4, 7, and 10), stems (pools 2, 5, 8, and 11), and needles (pools 3, 6, 9, and 12). A stringent filter was applied (false discovery rate [FDR]‐corrected *p*‐value <.001) to identify differentially expressed genes showing organ‐specific expression.

Once sequencing results were validated, a pairwise comparison analysis was performed pursuing two main objectives. On one hand, the identification of genes differentially expressed between WW plant and WD plants at the organ level, both in sensitive (1vs4, 2vs5, and 3vs6) and tolerant genotypes (7vs10, 8vs11, and 9vs12; see Figure 1a). On the other hand, the identification of constitutively expressed genes in WW plants at the organ level, but differentially expressed between sensitive and tolerant genotypes (1vs7, 2vs8, and 3vs9). Kal's *Z* test implemented in CLC Genomics Workbench v.10 (CLC Bio) was selected for statistical analysis to identify the differentially expressed genes (DEGs). Differential expression was considered for genes that satisfy the following criteria: (a) *p*‐value <.005; (b) fold change in count values >1.5; and (c) difference in count values >5.

### 2.6 Single enrichment and gene set enrichment analysis

Blast2GO results, including GO term annotation and KEGG pathway information, were used for functional analysis of differentially expressed genes. GO term enrichment was highlighted for every analysis (drought stress response as well as constitutive accumulation) by comparison between functionalities identified in genes significantly upregulated and downregulated. GO term enrichment was analyzed by using FatiGO (Al‐Shahrour et al., 2007) implemented in the OmicsBox software. Significant enrichment of GO terms was considered for *p‐*values <.05. Venn diagrams were drawn using Venny 2.1 (Oliveros, 2015). KEGG pathway enrichment analysis was carried out using GSEA software (Subramanian et al., 2005). All genes preranked according to Kal's statistics were included in order to analyze every comparison as a global system. Analyses were run with 1,000 permutations of gene sets. Pathways with *p‐*values <.05 were considered positively or negatively correlated.

### 2.7 Validation by quantitative real‐time PCR (qRT‐PCR)

**FIGURE 2:**
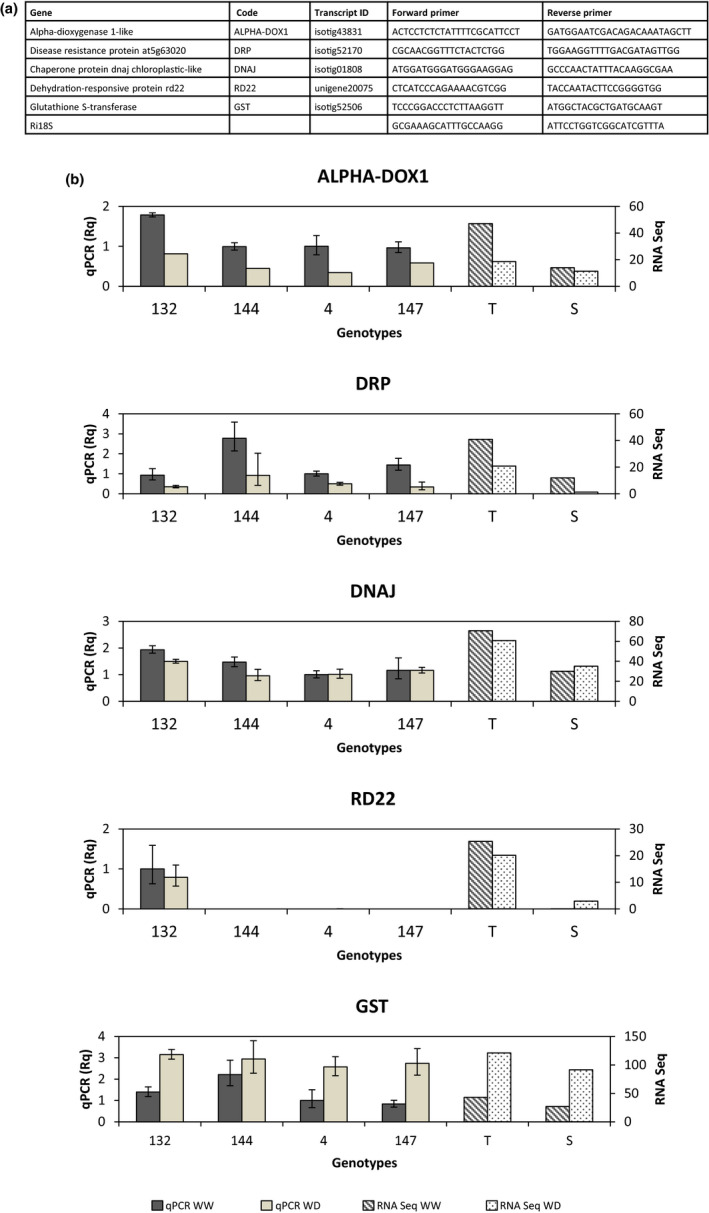
Validation of sequencing data by qRT‐PCR. (a) Primer sequences for qRT‐PCR. (b) Relative quantification (Rq) by qRT‐PCR (black and gray bars) and RNA‐seq normalized expression values (hatched and dotted bars) of five DEGs identified: alpha‐dioxygenase 1 (ALPHA‐DOX1), disease resistance protein at5g63020 (DRP), chaperone protein dnaj chloroplastic (DNAJ), dehydration‐responsive protein rd22 (RD22), and glutathione S‐transferase (GST). T: tolerant genotypes (132, 144); S: sensitive genotypes (4, 147)

In order to validate the transcriptomic study, expression analysis of a set of five DEGs was analyzed by real‐time qRT‐PCR. Gene specific primers were designed using the NCBI Primer‐Blast Tool (http://www.ncbi.nlm.nih.gov/tools/primer-blast/); sequences and transcript IDs are listed in Figure 2a. The 18S rRNA transcript was used as internal control for quantitative transcript accumulation analysis. Synthesis of cDNA was performed from 500ng of total RNA using SuperScript III First‐Strand Synthesis System (Invitrogen) according to the manufacturer's instructions. Polymerase chain reactions were carried out in an Applied Biosystems 7,500 Fast Real‐Time PCR System (Applied Biosystems), using FastStart Universal SYBR Green Master (Rox; Roche). Three replicates for the three ramets (biological replicates) per genotype, and three technical replicates of each biological replicate were analyzed targeting the organ in which each gene showed the highest differential expression between samples. The reactions containing 12.5 or 25 ng cDNA, 500 nm forward primer, 500 nm reverse primer, and 1× SYBR Green Master were subjected to an initial step of 10 min at 95°C, followed by 40 cycles of 15 s at 95°C and 60 s at 60°C. A melting‐curve analysis was included to verify the specificity of each primer. Relative expression was calculated by the ∆∆Ct method (Ct = threshold cycle) using the Relative Quantification application, powered by the Thermo Fisher Cloud platform.

## 3 RESULTS

### 3.1 Differential response of *Pinus pinaster* genotypes to water stress

Phenotypic characterization of these plants has been described by Sánchez‐Gómez et al. (2017). Physiological analyses indicate that water deficit had a general negative impact on leaf photosynthetic performance and osmotic potential. Yet, the four studied genotypes displayed two contrasting physiological sensitivities to water deficit. While the genotypes 132 and 144 performed as drought‐tolerant genotypes, 4 and 147 performed as drought‐sensitive. For example, water deficit did not affected genotypes 132 and 144 in terms of net photosynthetic rates, while it significantly decreased net photosynthetic rates of genotypes 4 and 147. This pattern of variation among clones was observed in other variables related to photosynthetic performance (i.e., stomatal conductance and effective quantum efficiency of photosystem II), as well as to growth rates. Nevertheless, the studied genotypes did not differ in osmotic adjustment (Sánchez‐Gómez et al., 2017).

### 3.2 Sequencing and generation of an improved *Pinus pinaster* reference transcriptome

Sequencing of the 12 libraries yielded a total of 2,416,362 reads, with an average length of 352 nucleotides before postprocessing, which were mapped against the ProCoGen reference transcriptome containing 191,544 transcripts. Unmapped reads (approximately 10%–17%) were used for de novo assembly, allowing the identification of 8,700 newly assembled transcripts longer than 200bp, which were added to the reference transcriptome to obtain the “*Pinus pinaster* drought response transcriptome” (PpDR transcriptome).

Approximately 54.5% of transcripts included in PpDR transcriptome were annotated. From 200,244 transcripts, blast results were found for 79,290 sequences comparing against protein sequences from model species from RefSeq database and for 29,539 additional sequences that showed similarity with protein sequences inferred from conifer genomes; however, a total of 91,415 transcripts could not be annotated, which may largely correspond to 5′ or 3′ non‐coding sequences, non‐coding transcripts, or putative pseudogenes considering the abundance of this type of transcripts observed in conifer genomes (Buschiazzo, Ritland, Bohlmann, & Ritland, 2012). The total number of transcripts included in the final PpDR transcriptome (200,244) is very high compared to the number of unigenes expected in a conifer genome (50,172 in *P. taeda* 2.0 (Neale et al., 2014, Zimin et al., 2017), 71,117 in *Pinus lambertiana* 1.5 (Crepeau, Langley, & Stevens, 2017; Stevens et al., 2016), 58,587 in *P. abies* 1.0 (Nystedt et al., 2013), or 102,915 in *P. glauca* 3.0 (Warren et al., 2015)), despite the large number of different unrelated sequences included in the assembly from different studies. Thus, an important degree of redundancy is expected and could be attributed to technical (i.e., frameshift errors associated with the different sequencing technologies (Luo, Tsementzi, Kyrpides, Read, & Konstantinidis, 2012)) and biological origins (i.e., splicing variants, large gene families observed in conifers (Ahuja & Neale, 2005) and to the high genetic diversity observed in *P. pinaster* (Mariette et al., 2001)). Considering this redundancy, multimapping was avoided in successive analysis, considering only the best match as the most probable for expression analysis.

Significant similarity to known domains was found for 92,499 transcripts, and 62,714 were annotated with 456,783 GO terms. Most of the assignments belonged to the biological process category (53.1%), while 27.5% and 19.4% of the GO terms were related to cellular component and molecular function categories, respectively. Additionally, 13,283 transcripts were annotated with 1,001 EC (Enzyme Commission) numbers and assigned to 145 KEGG pathways.

### 3.3 Mapping and identification of differentially expressed genes

Filtered reads from each library were independently mapped to the PpDR transcriptome. A total of 73,246 transcripts with at least one read in any of the cDNA libraries were detected. Roots showed the highest number of transcripts (>25,000), followed by stems and needles (Table 1).

**TABLE 1:** Summary of *Pinus pinaster* sequencing data trimming, number of mapped, and non‐redundant reads

Genotypes	Drought‐sensitive genotypes (4 and 147)						Drought‐tolerant genotypes (132 and 144)					
Treatment	Control (WW)			Water deficit (WD)			Control (WW)			Water deficit (WD)		
Organs	Roots	Stems	Needles	Roots	Stems	Needles	Roots	Stems	Needles	Roots	Stems	Needles
Library Number	1	2	3	4	5	6	7	8	9	10	11	12
Raw reads	194,839	130,159	191,484	219,310	150,111	181,699	243,036	179,117	175,323	253,378	228,029	269,877
Rejected by SeqTrimNext	17,885	26,510	38,202	25,248	29,895	35,773	28,793	29,053	32,293	38,837	38,592	54,587
Low complexity	6,336	2,506	2,921	6,888	2,801	2,899	7,265	3,034	3,011	7,420	4,038	4,180
Contaminated	1,480	2,506	2,626	1,341	1,920	4,069	1,485	1,334	3,745	1,849	1,538	6,375
Size	9,145	15,884	25,173	14,836	19,075	22,499	16,701	19,120	19,661	23,316	25,187	34,047
Empty insert	924	5,614	7,482	2,183	6,099	6,306	3,342	5,565	5,876	6,252	7,829	9,985
Reads after SeqTrimNext processing	176,954	103,649	153,282	194,062	120,216	145,926	214,243	150,064	143,030	214,541	189,437	215,290
Number of mapped reads	138,318	88,050	128,626	161,454	101,633	122,008	172,558	126,278	120,325	176,117	158,795	180,636
Non‐redundant reads	26,588	18,697	20,071	27,690	21,808	19,421	30,570	22,310	18,208	33,672	23,758	21,662

To preliminary validate the quality of sequencing data, a differential expression analysis was carried out using three data sets generated grouping all data by organ. The results clearly highlight organ‐specific expression patterns. The use of a highly restrictive threshold (FDR *p* <.001) led to identify a reduced group of transcripts with strong organ specificity. Needles showed the largest group of specific genes (99), followed by roots (25) and stems (8). Single enrichment analysis based on the needle‐specific genes showed a significant enrichment in GO terms related to biosynthetic and lipid metabolic processes, photosynthesis, and generation of precursor metabolites and energy, all of them functionalities associated with this organ, which support the quality of sequencing. Once sequencing was validated with a preliminary differential organ‐specific expression analysis, pairwise comparison was performed to analyze differential expression analysis in response to water stress. Thus, a total of 6,215 DEGs were identified (Kal's *Z* test, *p* < .005). Pairwise comparison of normalized read counts allowed detection of significant variation between WW‐ and WD‐sensitive and WW‐ and WD‐tolerant genotypes (i.e., 3,482 and 1,723 transcripts, respectively). Additionally, comparison between WW plants from sensitive and tolerant genotypes revealed a total of 2,993 transcripts accumulated in control conditions at different levels.

**FIGURE 3:**
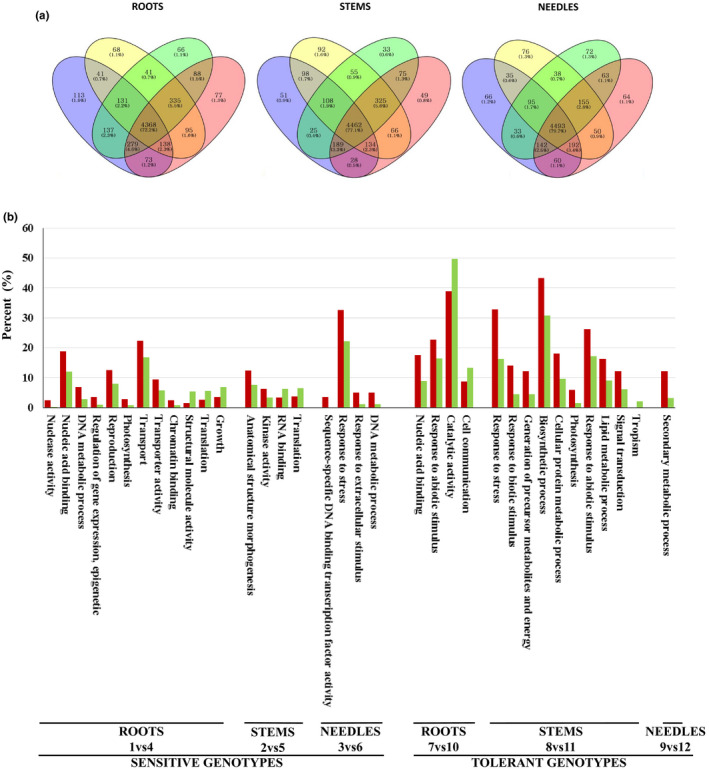
Gene expression analysis of *Pinus pinaster* genotypes under different water regimes. (a) Venn diagrams of 6.215 differentially expressed genes at the organ level. WW‐S: well‐watered sensitive plants. WD‐S: water deficit sensitive plants. WW‐T: well‐watered tolerant plants. WD‐T: water deficit tolerant plants. (b). Single enrichment analysis of differentially expressed genes in sensitive and tolerant genotypes, classified according to each GO terms. Red and green bars represent the percentage of GO biological process terms with upregulated and downregulated genes showing significant enrichment (*p* < .05), respectively

Venn diagrams of the 6,215 differentially expressed genes showed gene distribution in sensitive and tolerant plants in response to different water regimes analyzed at the organ level (Figure 3a). This analysis allowed identification of sensitive‐ and tolerant‐specific genes. The number of genes exclusively expressed in sensitive versus tolerant plants differed according to the organ analyzed: Roots and needles showed lower number of genes in sensitive plants (222 and 177, respectively) than in tolerant plants (231 and 199, respectively), while the opposite was observed in stems (241 and 157 genes in sensitive versus tolerant plants, respectively). Although the percentage of this class of genes observed in stems and needles of WD‐sensitive and WD‐tolerant plants was similar (79% in stems of both types of plants and 63% and 64% in needles of sensitive and tolerant plants, respectively), roots of WD‐tolerant plants showed a significant higher percentage tolerant‐specific genes than WD‐sensitive plants (71% and 49%, respectively). It is important to highlight that in all organs but stems, sensitive‐specific genes showed broader functional diversity than tolerant‐specific genes.

Comparison between organs also allowed the identification of DEGs shared between genotypes that showed similar trends (upregulation, downregulation, or nonsignificant variation in response to water stress; Table 2). Genes specifically upregulated or downregulated in a single organ were the most common trend observed for both sensitive and tolerant genotypes. Only two transcripts encoding a RING‐H2 finger protein (isotig37224) and a NAC transcription factor (unigene10311) were upregulated, while one transcript encoding a high‐affinity nitrate transporter (unigene30176) was downregulated in all organs of WD‐sensitive genotypes. When analyzing the organs of WD‐tolerant genotypes, we found no DEGs sharing the same expression trend. Two genes, a suppressor protein SRp40 (isotig43193) and a D‐tyrosyl‐tRNA deacylase (unigene27014), were highly upregulated in all organs of WW‐tolerant genotypes.

**Table 2:**
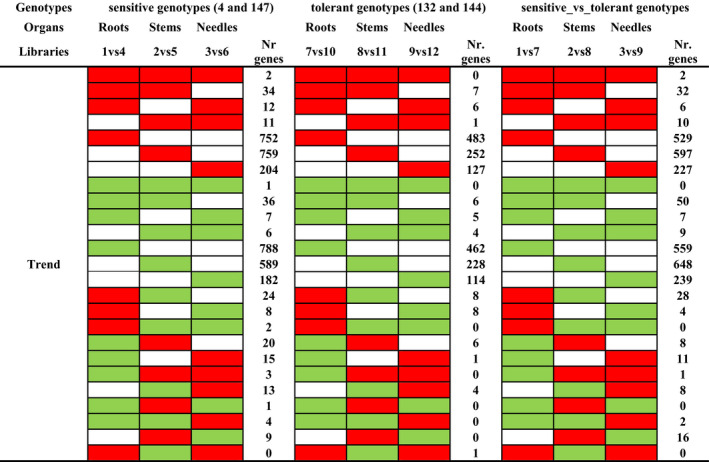
Main trend profiles followed by differentially expressed genes in water stressed sensitive and tolerant plants (WW versus WD) and between control plants (WW‐sensitive versus WW‐tolerant)

### 3.4 Functional analysis of genes differentially expressed in roots, stems, and needles in response to drought

Pairwise comparisons from sensitive and tolerant genotypes at the organ level allowed the identification of DEGs associated with water stress response. Sensitive genotypes showed higher number of DEGs than tolerant genotypes in all organs studied. Thus, 834, 839 and 264 genes were upregulated in sensitive genotypes, whereas 513, 266 and 140 DEGs were upregulated in tolerant genotypes in roots, stems, and needles, respectively. Downregulated genes followed similar trends in roots, stems, and needles of sensitive (875, 675, and 216) and tolerant (480, 251, and 131) genotypes, according to normalized read counts. Functional enrichment analysis between upregulated and downregulated genes highlighted GO terms significantly associated with different functions in each organ from tolerant and sensitive genotypes (Figure 3b).

Roots from sensitive genotypes showed a significant number of upregulated genes involved in nucleic acid binding and transport activity in response to water stress. In contrast, genes involved in growth, translation, and structural molecule activity were associated with downregulated genes. Lower number of GO terms was found significantly enriched in tolerant genotype roots, including upregulated genes involved in the response to abiotic stimulus and nucleic acid binding, as well as downregulated genes involved in catalytic activity and cellular communication (Figure 3b). KEGG analysis of genes encoding for enzymes leads to significant enrichment in different pathways. In roots of sensitive genotypes, the most significant pathways with upregulated genes were pantothenate and CoA biosynthesis, isoquinoline alkaloid biosynthesis, and styrene degradation, whereas phenylpropanoid biosynthesis, glutathione metabolism, and methane metabolism were the most significant pathways with downregulated genes. In roots of tolerant genotypes, flavone and flavonol biosynthesis, carotenoid biosynthesis, and pyruvate metabolism were the most significant pathways with upregulated genes, while amino sugar and nucleotide sugar metabolism and pyrimidine metabolism pathways included downregulated genes.

Stems of sensitive genotypes showed upregulated genes involved in anatomical structure and morphogenesis and kinase activity, while downregulated genes associated with translation and RNA binding (Figure 3b). Higher number of GO terms enriched in upregulated genes was observed in stems of tolerant genotypes, which were involved in biosynthetic process, response to stress, response to abiotic stimulus, lipid metabolic process, and signal transduction. The single function only represented by downregulated genes was tropism. Gene set enrichment analysis using GSEA highlighted significant upregulation of genes encoding enzymes from the pentose phosphate pathway in sensitive and tolerant genotypes. In addition, starch and sucrose metabolism pathways included upregulated genes, while fructose and mannose metabolism, as well as glycine and alanine pathways, showed downregulated genes in sensitive plants. Numerous KEGG pathways, such as glycine pathways phenylalanine metabolism, included upregulated genes in tolerant genotypes, while sphingolipid metabolism, arachidonic acid metabolism, and n‐glycan biosynthesis pathways showed downregulated genes.

Finally, only a few GO terms were significantly enriched in needles. We found upregulated genes involved in response to stress and extracellular stimulus, as well as sequence‐specific DNA‐binding transcription factor activity in sensitive genotypes, while in secondary metabolic process in tolerant genotypes (Figure 3b). Also, a few KEGG pathways were significantly enriched, showing different trends. In sensitive genotypes, arginine biosynthesis, glyoxylate, and dicarboxylate metabolism, as well as alanine, were the most significant pathways with upregulated genes, while amino sugar and nucleotide sugar metabolism, streptomycin biosynthesis, and butanoate metabolism were pathways with downregulated genes. No significantly enriched pathways with downregulated genes were observed in tolerant genotypes. However, numerous KEGG pathways were significantly upregulated, such as pantothenate and CoA biosynthesis, cutin, as well as cysteine and methionine metabolism.

**FIGURE 4:**
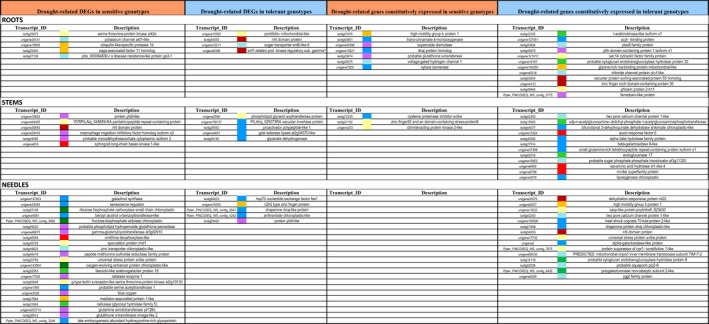
Drought‐responsive genes out of the 30 highest expressed genes in roots, stems, and needles of sensitive and tolerant genotypes. Differentially and constitutively expressed genes have been included. The colors indicate the different functionalities of the genes: structural and metabolic protection (dark blue), transport (light blue), signaling (light yellow) transcription factor and other regulator proteins (orange), detoxification (violet), cell wall component (light green), photosynthesis (dark green), plant growth regulators (light red), and hormone response (dark red)

Cluster analysis of co‐expressed genes in sensitive and tolerant genotypes allowed identification of groups of differentially expressed genes sharing expression patterns at the organ level (Table 3). 624 and 370 genes were upregulated, while 439 and 270 downregulated in roots; 709 and 192 genes upregulated, while 441 and 115 downregulated in stems; and 238 and 110 genes upregulated, while 146 and 74 downregulated in needles from sensitive and tolerant genotypes, respectively. Analysis of the 30 highest upregulated or downregulated genes classified according to their expression trend allowed identification of several enriched functionalities (Figure 4). Similar number of drought‐related genes was identified among the top‐30 highest upregulated DEGs in roots of both tolerant and sensitive genotypes subjected to water stress, which were involved in transport and signaling in all genotypes while in post‐transcriptional regulation (clustered as transcription factor and other regulator proteins) and in hormone response in sensitive and tolerant plants, respectively. Stems of sensitive and tolerant plants also showed similar number of highly upregulated drought‐related genes in response to water stress (Figure 4). However, in stems of sensitive plants these genes were involved in detoxification, hormone response, signaling, and plant growth regulation, while in tolerant plants they were mainly involved in lipid metabolism (classified as structural and metabolic protection). The analysis of the highly upregulated DEGs in needles showed a significant higher number of drought‐related genes in sensitive than in tolerant plants (Figure 4). They were mainly enriched in DEGs associated with a wide range of functions: detoxification (seven genes), structural, and metabolic protection (five genes), as well as photosynthesis (involved in Calvin and Benson cycle and photosystem II), signaling, cell wall remodeling, and transport. In contrast, only five out of the 30 highly upregulated DEGs were drought‐related in tolerant plants, mainly associated with structural and metabolic protection (three genes).

**Table 3:**
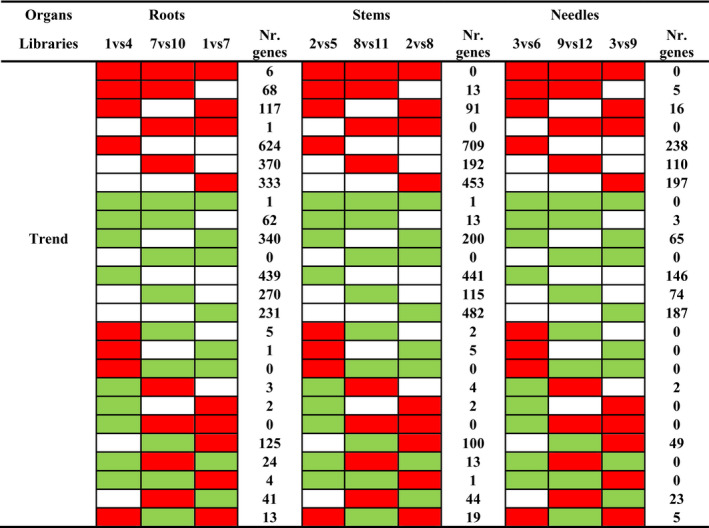
Main trend profiles followed by differentially expressed genes at organ level

### 3.5 Functional analysis of genes differentially expressed in WW plants at the organ level. Analysis of DEGs constitutively expressed

Comparison of normalized read counts between WW‐sensitive and WW‐tolerant genotypes allowed identification of DEGs in roots, stems, and needles. A total of 601, 666, and 267 were upregulated, while 638, 745, and 275 were downregulated in roots, stems, and needles, respectively. Gene ontology analysis revealed that highly upregulated genes in WW organs of sensitive and tolerant genotypes belong to different functional categories. Cell differentiation, secondary metabolic process, cell growth, and anatomical structure morphogenesis were enriched processes in WW roots of sensitive plants, whereas cellular metabolic process and enzymatic activities (kinase and hydrolase activities) were found in WW roots of tolerant plants. WW stems of sensitive genotypes were also enriched in secondary metabolic processes as well as in generation of precursor metabolites energy, while kinase and hydrolase activities, cell growth, and nucleotide binding GO terms were mainly associated with WW stems of tolerant plants. Finally, WW needles of sensitive plants were enriched in nucleotide binding, transferase activity, and lipid metabolic processes, while regulation of biological process, translation, and structural molecule activity was mainly found in WW needles of tolerant plants.

GSEA of roots and stems showed a common pattern of pathways significantly enriched, with: secondary metabolism, including flavonoid biosynthesis, glyoxylate, and dicarboxylate metabolism and phenylpropanoid biosynthesis upregulated pathways in roots; and oxidative phosphorylation, methane metabolism, and stilbenoid pathways as significantly upregulated in stems of WW‐sensitive plants. In WW‐tolerant plants, pantothenate and CoA biosynthesis as well as pyrimidine and galactose metabolism were upregulated in roots, while N‐glycan and lysine biosynthesis, and galactose metabolism were upregulated in stems. In WW needles of sensitive plants, biosynthesis or metabolism of different compounds, for instance, aminoacyl‐tRNA, butanoate, and phenylalanine was significantly enriched; however, none of significant pathway was enriched in WW‐tolerant plants. According to expression trends, 231, 482, and 187 genes were constitutively upregulated in roots, stems, and needles of sensitive plants, while 333, 453, and 197 genes were constitutively upregulated in roots, stems, and needles of tolerant plants, respectively (Table 3). Among the top‐30 highest constitutively upregulated genes, seven were drought‐related in roots of sensitive plants, involved in structural and metabolic protection, detoxification, transport, and regulation, while 12 in roots of tolerant plants covering more processes such as cell wall remodeling, transport, detoxification, and structural and metabolic protection, hormone response, signaling, and transcriptional regulation (Figure 4). In stems, only three out of the 30 highest constitutively upregulated genes were drought‐related in sensitive genotypes, involved in signaling and structural and metabolic protection, while 12 in tolerant plants involved in structural and metabolic protection, cell wall remodeling, transport across membranes, and plant growth regulation. In needles, none of the 30 highest constitutively upregulated genes identified in sensitive plants was drought‐related, while in tolerant plants were identified 15, classified in different functional groups: transport, structural and metabolic protection, cell wall reorganization, signaling, hormone response, and regulation (grouped as transcription factor and other regulator proteins).

It is important to highlight that six upregulated genes in roots of WW‐tolerant genotypes (Table 2) were also upregulated in roots from both types of genotypes in response to water stress: a LHY protein isoform X1 (unigene28840); a chaperone regulator 6 isoform X2 (unigene796); a putative calcium‐binding protein CML25 (isotig33124); two transcripts (isotig42238 and isotig75236) showing homology with a transposon type‐TNT 1–94; and finally, a *P. taeda* protein‐coding gene of unknown function (isotig31114).

### 3.6 Gene expression analysis by quantitative real‐time PCR

Expression analysis of five DEGs was analyzed on three ramets from each of the four genotypes by qRT‐PCR, in order to validate this study. Alpha‐dioxygenase 1 (ALPHA‐DOX1) and disease resistance protein at5g63020 (DRP) were analyzed in roots, and chaperone protein dnaj chloroplastic (DNAJ), dehydration‐responsive protein rd22 (RD22), and glutathione S‐transferase (GST) in needles of sensitive and tolerant plants were grown under different water regimens. The relative quantification of all these DEGs showed results in agreement with earlier transcriptomic analysis (Figure 2b).

## 4 DISCUSSION

This study provides new information about molecular strategies underlying differential response to a moderate water deficit intensity of roots, stems, and needles from four *P. pinaster* F_1_ progenies with contrasting responses to drought (de Miguel et al., 2012, 2014, 2016). Since plant response to drought stress is regulated by intensity, duration, and rate of progression of imposed drought (de Miguel et al., 2012; Pinheiro & Chaves, 2011), it was important to confirm that the effect of moderate drought in sensitive genotypes (4 and 147) was more intense than in tolerant genotypes (132 and 144). Moderate intensity of water deficit caused higher reduction of photosynthetic rate, stomatal conductance, and effective quantum efficiency of photosystem II in sensitive than in tolerant genotypes (Sánchez‐Gómez et al., 2017). Sensitive genotypes showed the highest water use efficiency under drought. This was associated with reduced transpiration water losses due to lower stomatal conductance rather than increased photosynthetic rates (Sánchez‐Gómez et al., 2017). In fact, increased water use efficiency under drought was coupled with poor photosynthetic performance in these sensitive genotypes. This finding is not surprising since high water use efficiency is not always correlated with fitness components such as growth and survival (Condon, Richards, Rebetzke, & Farquhar, 2004), and previous studies from natural ecosystems are inconsistent on the adaptive value of WUE for drought tolerance (Moran, Lauder, Musser, Stathos, & Shu, 2017; Nicotra & Davidson, 2010).

In this study, transcriptomic analysis of drought response at the organ level was based on pooled samples of sensitive and tolerant plants grown under WW and WS conditions. As previously described by Gonzalez‐Ibeas et al. (2016), although the lack of replicates in the bulked segregant transcriptomic analysis may hamper accurate identification of the low‐abundance differentially accumulated transcripts, this study was designed to provide a general trend of different biological processes underlying drought responses between tolerant and sensitive genotypes at the organ level. It is important to highlight that Assefa, Vandesompele, and Thas (2020) have recently validated the usefulness of RNA sample pooling strategies in RNA‐seq experiments using simulated RNA‐seq as well as empirical datasets. RNA‐seq based on pooled samples allowed reduction of the within‐group variability, enabling detection of biological effects with a small number of pools, as well as an associated reduction library preparation and sequencing costs.

Comparison between sensitive and tolerant genotypes revealed that the latter showed higher levels of transcriptional activity in the three organs but in WW needles (Table 1). However, the number of DEGs associated with water stress response was significantly higher in all organs from sensitive genotypes. Although the lowest number of DEGs was detected in the needles of both genotypes, they were significantly enriched in GO terms related to “response to stress,” as expected. These results may point to a basal activation of stress‐responsive mechanisms in tolerant genotypes that allow them to rapidly face frequent droughts. Other studies have also described sensitive genotypes that exhibit hyper‐response to drought stress compared to tolerant genotypes (Janiak et al., 2018; Muthusamy, Uma, Backiyarani, Saraswathi, & Chandrasekar, 2016; Pucholt, Sjödin, Weih, Rönnberg‐Wästljung, & Berlin, 2015; Yates et al., 2014 ; You et al., 2019), indicating absence of some stress avoidance mechanisms in these sensitive genotypes that attenuate drought effects.

Functional analysis of genes exclusively expressed in sensitive and tolerant plants also supported this hypothesis, which highlights the differences with higher number of genes involved in primary metabolic process, nitrogen compound metabolic process, and different GO terms associated with response to stress mainly in roots and needles of tolerant plants while higher functional diversity identified in sensitive genotypes. The presence of specific cell signaling processes in stems from tolerant genotypes, which were not present in the sensitive ones, may indicate an induction of organ‐specific transfer of information, which may be involved in response of tolerant genotypes to cope more effectively with drought fluctuations and its effects.

The analysis of specific pathways revealed interesting differential behavior between sensitive and tolerant genotypes. Thus, different pathways related to flavone and flavonol biosynthesis or carotenoid biosynthesis showed significant upregulated genes in roots of tolerant versus sensitive genotypes in response to water stress. Flavonoids play different molecular functions in stress protection, including inhibition of polar auxin transport, that interferes with hormone signaling (Dao, Linthorst, & Verpoorte, 2011), and antioxidant defense (Gill & Tuteja, 2010). Flavonoids accumulate at the site of lateral root formation in drought‐stressed Arabidopsis plants, which could suggest a positive effect on lateral root formation (Shojaie, Mostajeran, & Ghannadian, 2016) also found in poplar (Dash et al., 2017). Additionally, biosynthesis of carotenoids, which are precursors of abscisic acid (ABA) with functional roles as light harvesters, photoprotection, and structure stabilization, increases in roots of maize and Arabidopsis under drought and salt stress (Li, Vallabhaneni, Yu, Rocheford, & Wurtzel, 2008; Ruiz‐Sola, Arbona, Gómez‐Cadenas, Rodríguez‐Concepción, & Rodríguez‐Villalón, 2014).

Only six genes showing higher expression levels in WW‐tolerant than WW‐sensitive plants, which were also upregulated in WD‐tolerant and WD‐sensitive plants, were detected in roots (Table 3). Among them, transcripts promote abiotic stress tolerance such as chaperone, protecting structures (Park & Seo, 2015), and a dormancy auxin family‐associated protein (hormone involved in protein modification, signal transduction, and in drought tolerance (Peleg & Blumwald, 2011)). In addition, a late elongated hypocotyl protein (LHY), a MYB‐type transcription factor commonly associated with circadian clock control (Sanchez,Shin, & Davis, 2011), was also identified. In Arabidopsis, mutations in this gene produced hypersensitivity to ROS‐generating agents, which indicates a function during detoxification processes (Park, Kwon, Gil, & Park, 2016). Finally, a putative calcium‐binding protein CML25, a Ca^2+^ sensor involved in regulating plant responses to abiotic stresses (Zeng et al., 2015), was also detected. This set of transcripts, involved in protection, signaling, and regulation of gene expression, could also contribute to maintain tolerant genotypes in a constant alert state.

Pre‐adaptation of tolerant genotypes was also observed when analyzing genes involved in drought response out of the top‐30 highest constitutively upregulated genes, with genes mainly acting in: structural protection, such as osmoprotectants, aquaporins, chaperones, late embryogenesis abundant and heat shock proteins; detoxification of reactive oxygen species (ROS); and cell wall metabolism and protection of subcellular structures (Le Gall et al., 2015) (Figure 4). Thus, roots of sensitive plants showed lower number of constitutively upregulated genes (mainly involved in ROS scavenging, hydrogen transport through membrane, regulation of protein folding and aggregation, and sugar metabolism and phenylpropanoid biosynthesis), versus the 40% of genes found in tolerant plants (redox regulation, osmotic adjustment, cell wall remodeling, and transport, as well as hormone response, signaling, and transcriptional regulation). Upregulation of xyloglucan endotransglucosylase hydrolase (XTH), which modulates the biding of xyloglucans, the major hemicellulosic polymer, to cellulose, may be involved in cell wall remodeling. Many abiotic stresses lead to an increase in XTH gene expression (reviewed by Tenhaken, 2014). Considering that lipids are the major cell membrane components and a source for signaling molecules, upregulation of sec14 cytosolic factor family protein, a phosphatidylinositol/phosphatidylcholine transfer protein located in the Golgi membrane, that functions as signal precursor inducing stress‐responsive genes, phospholipids, and galactolipids (Liu et al., 2013), may promote membrane stability conferring stress tolerance (Larsson, Nyström, & Liljenberg, 2006). Additionally, ABA‐regulated genes and a SCF E3 ligase (phloem protein 2‐b11), recently described as a negative regulator of drought response involved in ABA‐independent signaling pathway in Arabidopsis (Li et al., 2014), were observed to be constitutive and highly overrepresented.

In stems, only three out of the 30 highest constitutively expressed genes were associated with drought response in sensitive plants: a cysteine proteinase inhibitor, involved in regulation of proteolysis (Kidrič, Kos, & Sabotič, 2014); and two genes involved in signaling, a zinc finger A20 and AN1 domain‐containing stress‐associated protein 8‐like (whose overexpression in rice reduced stress‐induced injuries such as chlorosis and cell death, improving recovery from stress (Vij & Tyagi, 2006)), and a CBL‐CIPK involved in decoding Ca^2+^ signals from calcineurin B‐like proteins. Evidence also indicates that a high number of CIPKs participate in various stress responses as well as in other ABA responses (Mao et al., 2016). As in roots, the number of constitutively expressed genes associated with drought response also increases up to 12 in stems of tolerant plants, with genes involved in protection, such as beta‐galactosidase 8 transcript, which may be associated with break of cell wall polysaccharides to direct sugars to cytoplasm to maintain of cell turgor under water loss (Gupta, Rai, Gayali, Chakraborty, & Chakraborty, 2016). Upregulation of components of the Ca^2+^‐permeable channel and the sugar phosphate/phosphate translocator (Jarzyniak & Jasiński, 2014) could be associated with the enhancement of the transport of these compounds across membranes. Cell wall reorganization (endoglucanase 17 and UDP‐N‐acetylglucosamine‐dolichyl‐phosphate N‐acetylglucosamine‐phosphotransferase genes) and adjustment of membrane lipids (alpha/beta hydrolase family protein and chloroplastic lipoxygenase) were also upregulated, considering that the two latter genes are also involved in signaling (Bae et al., 2016; Hamiaux et al., 2012). Upregulation of bifunctional 3‐dehydroquinate dehydratase/shikimate chloroplastic, involved in biosynthesis of aromatic amino acids and different aromatic secondary metabolites (alkaloids, flavonoids, lignins, and aromatic antibiotics) with important roles in plant stress response, was also detected. Changes in leaf water content occur together with the inhibition of stem expansion. Auxin influences stem elongation and regulates the formation of the plant shoot architecture (Gallavotti, 2013). Highly upregulated genes involved in auxin regulation were identified, such as auxin response factor 6, a transcriptional activator that regulates the expression of auxin response genes (Liu et al., 2014), and an IAA‐amino acid hydrolase ILR1‐like 4, that hydrolyzes IAA‐amino acid conjugates to produce free IAA (Ludwig‐Müller, 2011). Among hormone‐regulated genes, upregulation of a component of SCF (COI1) coreceptor in plants stands out. It has been reported to be involved in JA perception and signal transduction (Larrieu & Vernoux, 2016).

However, the most significant difference was found in needles, where none of the 30 highest constitutively expressed genes were associated with drought response in sensitive plants, versus 15 genes in tolerant genotypes clustered into different relevant functional groups (Figure 4), among them, genes with protective functions: preservation of the correct folding of RNA molecules and proteins during stress (i.e., chaperone, heat shock cognate 70 kDa), osmolyte biosynthesis (an alpha‐galactosidase‐like protein), and osmotic regulation (i.e., an aquaporin, a YGGT family protein and a two‐pore calcium channel protein 1, also constitutively accumulated in stems of tolerant genotypes). Protective effect of chloroplast‐targeted chaperone protein DnaJ on photosystem II has been previously described in transgenic plants subjected to stress, in which chloroplast heat shock protein 70 was also identified as a partner of this chaperone (Kong et al., 2014). In this study, a plasma membrane intrinsic protein (PIP) gene was also constitutively expressed. This subfamily of aquaporins is involved in water transport and, depending on the member family, small solutes transport, playing an active role in drought stress response (Forrest & Bhave, 2007), regulating biotic stress responses, and involved in regulating root water uptake and transpiration rates (Afzal, Howton, Sun, & Mukhtar, 2016). Upregulation of two‐pore calcium channel protein 1 in needles and stems of tolerant plants may modify stomatal aperture and transpiration rate, as it is well known that ABA‐induced Ca^2+^ transporters modify cytosolic Ca^2+^ concentration, regulating, for example, stomatal aperture in guard cells (Song et al., 2008) and also highlight the upregulation of a group of genes involved in cell wall remodeling (a xyloglucan endotransglucosylase hydrolase and a polygalacturonase noncatalytic subunit 2‐like) as well as a group that included drought‐inducible genes, such as RD22 and a member of NHL family, which are mediated by ABA. All these results also support that tolerant genotypes exhibit permanent activation of mechanisms for cell protection and overexpression of stress pathways that pre‐adapt them to respond more efficiently and rapidly to water stress.

A limited number of DEGs associated with drought response were upregulated in roots and stems of sensitive and tolerant genotypes subjected to drought. However, and in contrast with the higher number of drought‐related genes constitutively highly upregulated in tolerant plants, 23 out of the top‐30 highly upregulated genes in needles of sensitive plants subjected to drought were associated with drought response, versus five genes in needles of tolerant plants. The drastic reduction of stomatal conductance, photosynthetic rates, and effective quantum efficiency of photosystem II observed in sensitive genotypes may reflect the high upregulation of genes with protective functions observed in needles. This includes genes involved in preservation of the correct folding of RNA molecules and proteins during stress (i.e., LEA protein), osmolyte biosynthesis (i.e., galactinol synthase and probable serine acetyltransferase 1), and a group of genes involved in cell wall remodeling (cellulase [glycosyl hydrolase family 5] and fasciclin‐like [arabinogalactan protein 16], which is regulated by ABA). Stomatal closure associated with drought results in changes of rates of photosynthesis due to the decreased CO_2_ availability and production of reactive oxygen species (ROS), such as superoxide radicals (Osakabe et al., 2014). Increased photorespiratory activity during drought is also accompanied by elevated levels of glycolate oxidase activity, resulting in H_2_O_2_ production. In this study, seven out of the 23 upregulated genes were associated with enzymes that detoxify active oxygen species, such as catalase, glutathione S‐transferase omega‐like 2, glutamine amidotransferase YLR126C, phospholipid hydroperoxide glutathione peroxidase, and gamma‐glutamylcyclotransferase At3g02910, a member of the peptide methionine sulfoxide reductase, and a blue copper protein (Figure 4). Also, three genes with photosynthetic function, two of them related to the Calvin and Benson cycle (ribulose bisphosphate carboxylase small chain chloroplastic, fructose‐bisphosphate aldolase chloroplastic) and a chloroplast protein that acts as an auxiliary component of the photosystem II (oxygen‐evolving enhancer protein chloroplastic‐like), were highly upregulated in needles of sensitive plants. Additionally, three genes were associated with signaling: G‐type lectin S‐receptor‐like serine threonine–protein kinase At2g19130, sporulation protein RMD1, and universal stress protein a‐like protein. Genes encoding proteins with USP domain are useful in stress signal perception and seem to promote drought tolerance (Sinha et al., 2016). Finally, a component of a multiprotein complex transcription factor (mediator‐associated protein 1‐like) and genes involved in polyamine metabolism (ornithine decarboxylase‐like) and plant growth regulation were also highly upregulated. In the case of the needles of tolerant plants subjected to stress, the five highly upregulated DEGs were mainly involved in cell protection: a chaperone protein DnaJ chloroplastic‐like and a Hsp70 nucleotide exchange factor FES1, both protecting folding of molecules under stress; an anthranilate chloroplastic‐like involved in osmolyte biosynthesis that contributed to osmotic adjustment and thereby enhanced drought stress tolerance in plants (Liu, Shen, & Huang, 2015); and a protein YLS9‐like involved in oxidative response. The remaining fifth gene was a transcription factor C2H2‐type zinc finger protein, which is involved in regulation of drought response (Kiełbowicz‐Matuk, 2012).

## 5 CONCLUSIONS

One of the most interesting outcomes of this study was the finding that drought‐tolerant genotypes expressed a high number of genes related to stress even before the water deficit took place as opposed with the drought‐sensitive genotypes. This finding indicates that constitutive expression of drought‐related genes, specifically hormone‐regulated genes, genes involved in signaling pathways, as well as those involved in stress protection, can provide functional advantages to cope with an eventual water deficit. Interestingly, a significant number of genes related to the Calvin and Benson cycle and regulation of photosystem II were highly expressed in the sensitive genotypes but not in the tolerant genotypes when subjected to water deficit conditions. Regardless of the high expression of these genes in sensitive plants subjected to drought, they show low efficiency response. This may indicate that the expression of these genes does not represent a molecular adaptive acclimation response to drought in sensitive plants. Other regulating mechanisms may be controlling their poorest physiological response under water deficit. Despite some genes related to osmoregulatory protection were highly expressed under water deficit in sensitive plants but not in tolerant plants, the observed pattern of variation in osmotic potential did not reflect significant differences across genotypes which exhibited similar ability to adjust osmotically to the experimental water deficit. Overall, these results suggest that genes that are constitutively expressed under nonlimiting water conditions might be more related to an active adaptive physiological response to stress conditions than those genes facultatively expressed under water deficit, which might reflect in part, constraints, restrictions, and alterations of the transcription process imposed by environmental stress.

## CONFLICT OF INTEREST

The authors declare that the research was conducted in the absence of any commercial or financial relationships that could be construed as a potential conflict of interest.

## AUTHOR CONTRIBUTION

**Nuria de María:** Data curation (equal); Formal analysis (equal); Investigation (equal); Methodology (equal); Writing‐original draft (equal); Writing‐review & editing (equal). **M. Ángeles Guevara:** Formal analysis (equal); Investigation (equal); Methodology (equal); Writing‐original draft (equal); Writing‐review & editing (equal). **Pedro Perdiguero:** Data curation (equal); Writing‐original draft (equal); Writing‐review & editing (equal). **M. Dolores Vélez:** Formal analysis (equal); Investigation (equal); Methodology (equal); Writing‐review & editing (equal). **J. Antonio Cabezas:** Formal analysis (supporting); Investigation (equal); Writing‐review & editing (equal). **Miriam López‐Hinojosa:** Formal analysis (supporting); Investigation (supporting); Methodology (supporting); Writing‐review & editing (supporting). **Zhen Li:** Data curation (supporting); Writing‐review & editing (supporting). **L. Manuel Díaz:** Formal analysis (equal); Methodology (equal). **Alberto Pizarro:** Formal analysis (supporting); Investigation (supporting); Methodology (equal). **J. Antonio Mancha:** Methodology (supporting). **Lieven Sterck:** Data curation (equal); Writing‐review & editing (supporting). **David Sánchez‐Gómez:** Investigation (equal); Methodology (supporting); Writing‐review & editing (equal). **Célia Miguel:** Investigation (supporting); Writing‐review & editing (supporting). **Carmen Collada:** Investigation (equal); Writing‐review & editing (equal). **Carmen Díaz‐Sala:** Investigation (equal); Writing‐review & editing (equal). **Maria‐Teresa Cervera:** Conceptualization (equal); Investigation (equal); Supervision (equal); Writing‐original draft (equal); Writing‐review & editing (equal).

## Data Availability

Sequences generated in this study were submitted to the NCBI Sequence Read Archive (SRA) database under BioProject accession number PRJNA590115 (SRA accession numbers from SRR11812379 to SRR11812390). Sequences, descriptions, and annotations of *P. pinaster* PpDR_transcriptome are available at the Figshare repository under the https://doi.org/10.6084/m9.figshare.12328979.
